# Semantic Factors Predict the Rate of Lexical Replacement of Content Words

**DOI:** 10.1371/journal.pone.0147924

**Published:** 2016-01-28

**Authors:** Susanne Vejdemo, Thomas Hörberg

**Affiliations:** Department of Linguistics, Stockholm University, Stockholm, Sweden; University of California, Irvine, UNITED STATES

## Abstract

The rate of lexical replacement estimates the diachronic stability of word forms on the basis of how frequently a proto-language word is replaced or retained in its daughter languages. Lexical replacement rate has been shown to be highly related to word class and word frequency. In this paper, we argue that content words and function words behave differently with respect to lexical replacement rate, and we show that semantic factors predict the lexical replacement rate of content words. For the 167 content items in the Swadesh list, data was gathered on the features of lexical replacement rate, word class, frequency, age of acquisition, synonyms, arousal, imageability and average mutual information, either from published databases or gathered from corpora and lexica. A linear regression model shows that, in addition to frequency, synonyms, senses and imageability are significantly related to the lexical replacement rate of content words–in particular the number of synonyms that a word has. The model shows no differences in lexical replacement rate between word classes, and outperforms a model with word class and word frequency predictors only.

## Introduction

Words are continuously being replaced in the languages of the world. But not all words are replaced at the same pace or for the same reasons. For example, Dahl [1] noted that, in the time since Latin, words for girl had been replaced far more in a handful of Romance languages than words for tree. What are the reasons behind whether a word will be replaced or not? How much faster are some words replaced than others? Recent research has shown that these questions can be partially answered by correlational statistical investigations of language data (see [2], [3], [4], [5], [6]). In a similar vein, the goal of this paper is to show that, in addition to frequency, semantic factors (namely synonyms, senses and imageability) predict the rate of lexical replacement of content words.

A relative rate of lexical replacement for a concept can be estimated by counting the number of times an original proto-language word is replaced or retained in its daughter languages (e.g., [1]; [2]). (Retention or absence of a word is operationalized as presence or absence of a cognate on a Swadesh list of primary word form. Naturally, the absence of a cognate on such a list does not mean that a cognate word is not present in the language with a slightly different meaning. In the rest of this text, *cognate* should be understood as *synonymous cognate* (also called *s-cognate*)–words that not only share a common ancestor, but also mean roughly the same thing at present.) Pagel et al. [2] calculated a relative rate of lexical replacement for the primary words (cf. primary designating expressions in [7]) of the 200 concepts of the Swadesh list, based upon data from Dyen, James & Cole [8] on the frequency of change of these concepts in Indo-European language varieties. As an illustration, Table 1 has the translation equivalents for the concept dirty and tongue in several Slavic and Germanic languages. Whereas this particular sample of languages has eight different cognate classes for dirty, all of the languages have a contemporary word that is a cognate of the Indo-European original word for tongue. (The exact cognate class categorizations can of course be discussed in all cases–for TONGUE, Darling Buck (1949:230) notes that another root, *sighwa, might also be involved, blended with *dnghwa.)

**Table 1:** Translation equivalents for the concepts dirty and tongue in some Slavic and Germanic languages. Whereas the words for DIRTY come from eight different cognate classes, the words for TONGUE are all a cognate of the Indo-European original word *dnghwa, and therefore come from one cognate class.

Language name	Language family	DIRTY		TONGUE	
		Words	Classes	Words	Class
Byelorussian	Slavic	BRUDNY	1	Jazyk	1
Slovak	Slavic	BRUDNY	1	Jazyk	1
Polish	Slavic	BRUDNY	1	Jezyk	1
Czech	Slavic	SPINAVY	2	Jazyk	1
Icelandic	Germanic	SKITUGUR	3	Tunga	1
Norwegian	Germanic	SKIDDEN	3	Tunge	1
Faroese	Germanic	SKITIN	3	Tunga	1
Danish	Germanic	BESKIDT	3	Tunge	1
Sorbian	Slavic	MAZANY	4	Jazyk	1
Slovenian	Slavic	UMAZANU	4	Jezik	1
Bulgarian	Slavic	MRESNO	5	Ezik	1
Serbocroatian	Slavic	PRLJAV	6	Jezik	1
Macedonian	Slavic	PRLAV	6	Jazik	1
German	Germanic	SCHMUTZIG	7	Zunge	1
English	Germanic	DIRTY	8	Tongue	1

If the sample size is enlarged to include all the Indo-European languages in Dyen, James & Cole [8], there are, in total, 46 cognate classes for dirty and but only 4 cognate classes for tongue, indicating that the former concept has been replaced much faster than the latter. Pagel et al.’s [2] measure of Lexical Replacement Rate is based on such data, but is also weighted by the language family relationships between languages. The rate thus measures relative diversity in the sample languages in the Swadesh list, and can be used to estimate the the average relative rate of lexical replacement.

Pagel et al. [2] found that both modern day word frequency and word class predict whether a concept is likely to retain or change its lexical inventory. Using regression modeling, they found that lemmatized corpus frequency and word class explains a large part of the variance in Lexical Replacement Rate, regardless of which language the frequency information is from (English, R = 0.69; Spanish, R = 0.69; Russian, R = 0.71; and Greek, R = 0.69: all *p*:s < .0001.) Concepts that are used more frequently in modern day corpora tend not to be replaced as often as less frequently used concepts. When controlling for frequency, the replacement rate is fastest for concepts usually expressed by prepositions and conjunctions, followed by adjectives, verbs, nouns, special adverbs, pronouns and finally numbers. (Word class division was done on the meta language English, and then assumed to be the same for all cognates in all the other languages. While this is most likely doable for Indo-European, it should be noted that the method might not be suitable for other language families, where word classes might be quite different.)

Building on Pagel et al, Monaghan [5] found that age of acquisition, and the correlated features of concreteness and phonological length (words that children learn first are typically for concrete objects, and are short), affected the rate of lexical replacement.

In this paper, we will argue that it is advisable to treat function words (such as preposition, conjunctions, adverbs, pronouns and numbers) and content words (nouns, verbs and adjectives) differently when seeking to understand rates of lexical replacement. We will evaluate the predictive power of several potential semantic factors behind the rate of lexical replacement for content words through correlation and multiple regression tests. We will first focus on the difference between content and function words and, once we have made the case that it is worthwhile to proceed and look only at content words, we will turn to their rate of replacement.

### Content and function words

When examining which factors affect rates of lexical replacement, there are good reasons to consider open and closed word classes separately. Open word classes host content words, such as tongue, stone, woman and closed word classes host grammatical (function) words such as and, but, three. Open word classes, especially nouns, get new members (e.g. when new objects need to be named), whereas new grammatical functions appear more rarely in a language. There is also a cognitive divide in the brain’s handling of content and function words. Whereas clinical patients suffering from expressive aphasias generally have problems in producing function words and morphosyntactic structure, patients with receptive aphasias are often unable to comprehend and select correct content words during speech production [9]. There are also clear differences in neurophysiological activity during the processing of function words in comparison to the processing of content words [10–12].

**Fig 1:**
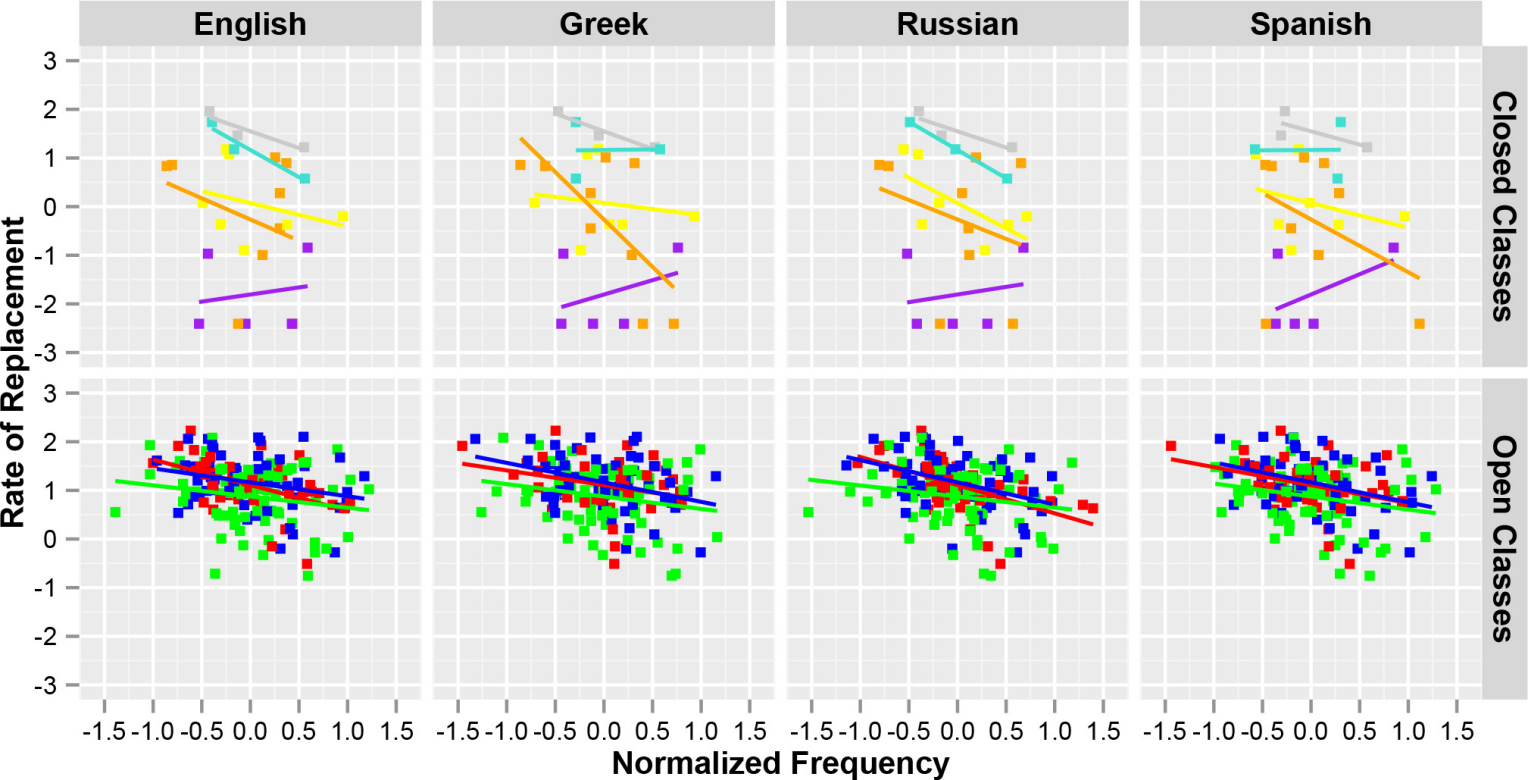
Lexical replacement rate as function of normalized frequency in English, Greek, Russian and Spanish, for concepts of open (red: adjectives, green: nouns, blue: verbs) and closed (yellow: adverbs, grey: conjunctions, purple: numbers, turquoise: prepositions, orange: pronouns) word classes, respectively.

The data from the Pagel et al.’s [2] study also suggests that concepts from open versus closed word classes behave different with respect to their rate of lexical replacement. The word classes present in Pagel et al.’s data belong to two distinctive groups: open (173 items: 40 adjectives, 58 verbs and 75 nouns.) and closed (27 items: 3 conjunctions (and, because, if), 3 prepositions (in, with, at), 5 numbers (one, two, three, four, five), 7 adverbs (here, there, how, where, when, what, not) and 7 pronouns (I, thou, he, we, ye, they, who).) word classes. Regression analyses conducted separately for each language in the Pagel study show that, for the closed class words, the variation in the lexical replacement largely depends on word class differences (56.1% in English, 51.6% in Spanish, 56.1% in Russian, and 54.4% in Greek; all *p*:s < .001) and too a much smaller degree on frequency differences (4% in English, 0.8% in Spanish, 5.6% in Russian, 1.8% in Greek; all *p*:s < .001). Concepts from open word classes, on the other hand, make up a much more homogenous group for which a substantially larger part of the variation in lexical replacement rate is left unexplained: regression analyses for open class words show that even when frequency and word class are taken together, they explain a much smaller portion of the variation (14.6% in English, 15.2% in Spanish, 16.2% in Russian, and 14.3% in Greek; all *p*:s < .0001). The differences in lexical replacement rates between open and closed word classes are illustrated in Fig 1. All figures in this paper was created using the ggplot2 package in the statistical software R [13,14].

We therefore suggest that the rate at which closed class items change is more related to idiosyncratic properties of the individual word. Closed class items are always further along the grammaticization continuum, and are therefore more abstract and general in meaning, more widely applicable and more frequently used [15]. Taken together, this suggests that the lexical replacement rate of closed class items is more dependent on diachronic processes of the constructions that those items frequently occur in. The lexical replacement rate of open class items, on the other hand, might therefore be less sensitive to diachronic changes of specific constructions, and more dependent on lexical semantic and pragmatic factors of those items. In the following, we will try to establish which some of those factors might be, using correlation and linear regression analysis.

## Materials and Methods

It must first be acknowledged that the factors that drive lexical replacement are probably very many indeed, and that their interplay no doubt is complex (see, e.g. Ladd et al. [6] for a review of studies on the interactions of factors such as e.g., lexical replacement rate, frequency and concreteness). It is also very likely that cultural considerations are very important for which words get replaced, and that these cultural considerations vary between speaker communities, and over time. In addition, specific semantic domains (such as body parts, kinship terms, colors etc.) probably have domain specific tendencies when it comes to likelihood of lexical replacement. Our investigation of lexical replacement takes none of this into account, and instead tries to investigate if it is possible to find evidence for domain-overriding generalizations of which factors can affect the rate of lexical replacement.

We will first present the motivations for including each factor, and how the factors were operationalized. Next, we will examine the results of the correlation and multiple regression tests.

This study investigates the relationship between semantic factors and the rate of lexical replacement of content words over and above that of frequency, word class and age of acquisition–three factors that others have found to impact lexical replacement rate (see [2,5]). These factors are: co-occurrence with other lexical items, as measured by average *mutual information*; *imageability*; likelihood of euphemisms as measured in terms of *arousal*; polysemy in terms of number of *senses;* and finally the number of *synonyms*. In the following, we present the motivations for including these factors and how they are operationalized to enable statistical testing. With the exception of the data on frequency and synonyms, all the data is taken from English, a possible caveat that we return to in the discussion.

### Word frequency and word class

Pagel et al.’s [2] findings clearly show that word frequency and word class predict the lexical replacement rate. We also include frequency and word class in our investigation. The Frequency data is taken from the average of the frequencies reported in Pagel et al. 2007 for English, Spanish, Russian and Greek. In order to increase comparability, the Pagel et al. data has been used whenever possible. Word frequency often emerges as a robust predictor in models predicting lexical entrenchment in memory, but it is far from clear what exactly a frequency count of word use in texts measures when it comes to human cognition. Baayen (2010) has shown that the simple assumption that frequency, understood as the number of repetitions of a word a speaker/hearer is used to (a “counter in the head”), represents activation levels or level of entrenchment, may be too simplistic. Baayen shows that in his lexical decision model, 90% of the variance in word frequencies is predictable from other lexical properties. He points out that although a frequency count is often statistically a strong predictor for, e.g. lexical decision experiments, a frequency count should in reality be understood as representing a wide range of lexical distributional properties such as contextual diversity, dispersion over different kinds of texts, the ratio of how often a word is written or spoken etc. With this understanding in mind, that frequency does not simply represent repetition, we will continue to use frequency so as to not conflate our model with ten or more additional variables, most of which might be difficult to obtain for our Swadesh list items.The word class data is also from Pagel et al. [2].

### Age of acquisition

Age of acquisition was found by Monaghan [5] to correlate with the rate of replacement. Like that author, we use data from Kuperman et al [16].

### Mutual information

As a frequency measure, Pagel et al. [2] considered only the word count, but we believe there might be good reason to also include a different kind of frequency measurement, namely the likelihood of co-occurrence with other words. It might be more difficult to replace a word that often co-occurs with other words in constructions than to replace a word which does not have such common co-occurrences (e.g., brother might occur often with sister, while to go might have no such steady lexical partner to anchor it). Any measure of strength of co-occurrence would thus be expected to be negatively related with the rate of replacement.

The likelihood of co-occurrence was operationalized by averaging the 20 highest Mutual Information neighbors of each item in the English BNC. *Mutual Information* is a co-occurrence measurement which give high values to two items that often co-occur (*salt* would have a high MI value with *pepper*) and low values to items to rarely co-occur (*salt* would have a low MI value with *dinosaur*). When MI for two words is calculated, the frequency of both words independently is taken into consideration and contrasted with how likely the words are to occur together. An estimate of the mutual information of items X and Y is defined as

$$MI(x,y)=\frac{\text{log}(n(xy)*N)}{n(x)*n(y)-ngramsize}/\text{log}\;2$$

where n(xy) is the frequency of co-occurrences of x and y, n(x) the frequency of x, n(y) the frequency of y, and ngramsize the size of the n-gram window under investigation. The Mutual Information data for the 100 million word British National Corpus was extracted from the http://corpus.byu.edu/bnc/ interface.

### Arousal

Another potential factor behind lexical replacement is the number of euphemisms of a concept. There is a substantial body of research on the effects of taboo and euphemism on lexical change. Linguistic taboos can certainly be very local, but can also be, if not universal, then at least very widespread [17,18]. Once a linguistic taboo exists, speakers have various strategies to avoid the offending word [19,20]. In this way, the euphemisms can lead to a great plurality of synonyms and accelerated lexical replacement. Burridge [21] writes that “very few euphemisms that have degraded [by association with taboo topics] into taboo terms come back from the abyss, even after they have lost their taboo sense. This promotes an ever-changing chain of vocabulary for words denoting taboo concepts”. Grzega [22] also notes that pejoration is an important factor in lexical replacement, and Pinker [23] has dubbed this effect "the euphemism treadmill". Linguistic taboo is not binary: concepts can be more or less taboo, and thus lead to more or less lexical replacement [21]. While it is often not difficult to point out clear cut cases of linguistic taboo (like the ever changing proper vocabulary for the taboo concepts of sperm or urine or vomit, for instance), there are also many cases of semantic detoriation where taboo is perhaps too strong a word. Grzega [22] gives the example of how words for the concept girl seem to be culturally charged and have to change often to avoid the unintended associations they repeatedly seem to evoke, even though few would say that girls are taboo in e.g. English speaking societies. If a measurement can be found for this "emotional charge" attached to concepts that engender euphemism, it would be expected to be positively related with the rate of lexical replacement: those concepts that have a higher emotional charge (maybe to die, woman) etc. and that lead to more euphemisms, will undergo word replacement faster than those that have a lower emotional charge (stone, to go).

The likelihood of a concept engendering many euphemisms is operationalized by another common measure in psychology: *arousal*. Arousal (together with Valence and Power) is measured by the Semantic Differential technique (pioneered by Osgood [24]) through questionnaires where speakers rate a word according to several different axes. A high arousal means that the word evokes more emotion in the participant than low emotion. This study uses data from Warriner et al.[25].

### Imageability

There is a marked difference between highly imageable (i.e. easy to picture in the mind) and less imageable concepts. Much psycholinguistic research has investigated the difference in brain processing of these two categories of concepts: Paivio [26] bases the dual coding theory of cognitive organization partly on the difference between processing of easily visualized and hard to visualize words. Crutch and Warrington [27] also write about the fundamental differences between the categories. Mårtensson et al. [28] show that nouns connected to sensory semantic (visual and otherwise) features are dealt with in different parts of the brain than those that do not. Work has been done on the differences between easily and not so easily imaged nouns [29] and likewise in verbs [30]. The link between imageability and lexical replacement, more specifically between low imageability and slow lexical replacement, was also raised in a pilot study [3] which examined the lexical replacement in word inventories for concepts in both Indo-European and Austronesian. Those concepts that are more easily imagined and pictured in the minds of the speakers (stone, in contrast to old) would be expected to undergo less word replacement. Any measure of imageability would thus have a negative relationship with the rate of replacement.

When measuring *imageability*, participants are asked how easy it is to form a mental image, when presented with a particular word. A closely related semantic feature to imageability is concreteness: participants are in this case asked how concrete a word is (see Hills and Adelman [31] for a discussion of how concreteness interacts with word learnability and usage). Monaghan [5] shows that concreteness correlates with the rate of lexical replacement, using data from Brysbaert et al [32]. This study uses imageability data from Cortese & Fugett [33], who have published imageability ratings from English speakers for many English words. The Cortese & Fugett imageability data and the Brysbaert et al. concreteness data for the Swadesh items correlate strongly (r = 0.88 *p* < .0001).

### Senses

The degree to which the primary word of a concept is polysemous and therefore has many different senses may also affect its lexical replacement rate. Words with many senses can be used in more different genres than words with few senses, and this might lead to a greater entrenchment which thereby might insulate a word somewhat from replacement (see [34] for a discussion on entrenchment). A measurement of the number of semantic senses should therefore be negatively related to the rate of replacement.

In the study, the number of *senses* was determined on the basis of the Wordnet English lexical database [35] where all lexical items are tagged with how many senses they have.

### Synonyms

There are finally good reasons to suspect that the number of synonyms a concept has is related to its lexical replacement rate. There is good evidence, from psychology and neurobiology, that words and meanings in the mind are stored in some kind of network structure ([36], chapter 10). In association tasks, where participants are asked to freely associate from a given stimulus word, the target word tend to be semantically related to the stimulus word in terms of coordination (words which cluster together semantically, like butterfly and moth, and often share a hypernym), collocation (words which are often found together with the stimulus, such as salt and water, bright and red), superordination (insect is elicited by butterfly) and synonymy (starved and hungry) [37]. In lexical decision tasks, in which subjects determine whether a stimulus word is a correct word or a nonsense word, response times are lower [38] and the neurophysiological response to lexical-semantic processing is reduced [39] when the target word is preceded by a semantically related word. Semantically related words therefore prime each other, indicating that they are co-activated during lexical access and thus psychologically related. Traugott and Dasher [34] write that the main driving force in regular semantic change is pragmatic, and that if a word is gained or replaced for a particular concept, it happens gradually. The pragmatic cognitive tool of inferencing is an important part in lexical replacement and change: if the meanings M1 and M2 are semantically related in some way, W2, which denotes M2, can through an inference come to denote M1. Concepts that that are more involved in more inferencing might thus be expected to be replaced more often.

Items with more semantic connections with other items could have a higher chance of replacement, since this would mean replacing the item with a nearby synonym would be easier. The number of synonyms of a concept should therefore be positively related to lexical replacement rate.

In the study, the number of *synonyms* that a word has is measured by counting the number of suggested synonyms in synonym dictionaries. For the English data, the synonyms came from the Oxford Pocket American Thesaurus [40] since this proved easy to automatically extract data from. However, synonym dictionaries do not contain only synonyms, but also common hyponyms and hypernyms–and also metaphorical synonyms. Some words have more metaphorical synonyms than others. In any case, all these semantic relations are all evidence for semantic connections, and for brevity “synonyms” will be used in this article to refer to them. In order to get a more balanced average synonym count for the underlying concept, we also gathered data from synonym dictionaries in four other Germanic languages: Swedish [41], Danish [42], German (http://www.woerterbuch.info/) and Dutch (http://synoniemen.net), and averaged the synonym count by weighing it first by the verbosity of the specific synonym dictionary (if the average number of synonyms given were 18, the number of synonyms for the specific word would be divided by 18) and then averaging the result over all five languages.

It will be important to bear in mind that several of these variables might not be independent from one another: several of them might be interrelated. Particular notice should be paid to how word class interacts with the different variables.

## Results and Discussion

The data sources for the independent variables in the model have been discussed in the previous section. The dependent variable, the Rate of Lexical Replacement comes from [2]. The data concerns 167 of the 173 items listed as open class words in [2]: the missing six items are listed as open class items, but are judged by us as being semantically close to closed class items (these are: all, few, many, near, other, some, that, this), and so have been excluded.

All statistical analyses were conducted with the statistical software R, primarily with the integrated stats package [13]. The Frequency, Senses and Age of Acquisition predictors were log transformed in order to have an approximately normal distribution. As an initial analysis, the correlations among all of the continuous variables were investigated. The correlation matrix with the Pearson correlations among all of the variables is shown in Table 2. As evident from the table, Lexical Replacement rate is significantly correlated with Frequency, Age of Acquisition, Synonyms, Mutual Information and Imageability.

**Table 2:** Correlation matrix of correlations between all variables in the study. P values for significance tests have been corrected for multiple comparisons using Holm correction.

	Rate	Log Frequency	Synonyms	Mutual-Information	Imageability	Arousal	Log Senses	Log AgeOfAcq
Rate	-	-.273**	.242*	-.281*	-.254*	.029	-.046	.255*
LogFrequency	-.273**	-	.381***	.221	-.208	-.046	.357***	-.482***
Synonyms	.242*	.381***	-	-.003	-.486***	.195	.592***	-.043
Mutual-Information	-.281*	.221	-.003	-	.506***	-.111	.031	-.367***
Imageability	-.254*	-.208	-.486***	.506***	-	-.071	-.369***	-.238
Arousal	.029	-.046	.195	-.111	-.071	-	.046	.097
LogSenses	-.046	.357***	.592***	.031	-.369***	.046	-	-.178
LogAgeOfAcq	.255*	-.482***	-.043	-.367***	-.238	.097	-.178	-

***: *p* < .0001

**: *p* < .01

* *p* < .05.

There are also high correlations among many of the dependent variables themselves (e.g., Synonyms and Senses, Mutual Information and Imageability). It is therefore unclear whether lexical replacement rate indeed is individually related to the variables at hand, or whether these relationships are mediated by the interdependencies among the dependent variables themselves.

In order to overcome this problem, data was analyzed using multiple regression modeling. This method models a normally distributed continuous variable, the outcome variable, as a linear combination of a set of independent or predictor variables. Importantly, the model estimates the individual relationships between each predictor and the outcome variable while controlling for the influence of all other predictor variables in the model by partialling them out. The model contains the continuous predictors shown in Table 2, together with the word class predictor (i.e., Word Class, Frequency (in log form, henceforth LogFrequency), Age of Acquisition (henceforth LogAgeofAcq), Imageability, Mutual Information, Synonyms, Senses (henceforth LogSenses), and Arousal).

A concern in linear regression is *overfitting* of the regression model. If the model contains too many predictors in relation to the sample size, the model coefficients might be overoptimistic and the model predictions will not generalize beyond the sample data (c.f., e.g., [43]). Overfitting was evaluated using bootstrap validation. Overall overfitting was estimated by calculating the shrinkage coefficients γ_0_ and γ_1_ on the basis of 10000 bootstrap samples, using the boot package [44]. The model is refit on each bootstrap sample and the observed values of the original data set is regressed against the predicted values of each bootstrapped model. γ_0_ and γ_1_ is then estimated as the mean of the intercepts and the slopes of the bootstrapped models. The coefficients did not differ significantly from the intercept and slope of the observed values regressed against the predicted values of the original model (i.e., 0 and 1, respectively), as evident by Z tests (γ_0_ = 0.41, Z = -0.92, *p* = .36; γ_1_ = 0.87, Z = 0.95, *p* = 0.34) (c.f. [45,46,47]).

A final concern in linear regression is (multi)collinearity, that is, the correlation between two or several predictors in the model. Collinear predictors may not individually account for the variance in the outcome variable. This in turn increases the standard errors of the coefficient estimates, and therefore reduces the confidence of those estimates. As shown in Table 2, The high correlations between individual predictors together with measures of the Variance Inflation Factor (max VIF: 5.9), which estimates correlations between an individual predictor and all other model predictors (cf., e.g., Harrell, 2010:65), indicates that multicollinearity might be a concern. Bootstrapping was therefore also used to test the significance in the individual predictors, independently of their standard errors. Predictor estimates were calculated on the basis of 10000 bootstrap samples, shown in Table 3. The predictor statistics of the bootstrapped model confirm the predictor effects in the original model in terms of both effect direction and significance (see Table 3), and therefore attests to the stability of the predictors.

**Table 3:** β coefficients and inferential statistics of the original and the bootstrapped model. The table also includes 95% point wise confidence intervals for the coefficients, based on the 0.025 and 0.975 quantiles of the coefficient estimates of the 10000 bootstrap samples. The table also includes ΔR^2^ for each predictor, that is, the proportion of variance of Lexical Replacement Rate explained by each predictor, over and above that of all other predictors in the model. For technical reasons, the Word Class variable, which has three values (verb, noun or adjective), is represented as three different binary variables: Word class: Noun, Word class: Verb, Word class: Adjective, and the last of these is not entered into the model since its information is already there: if something is not a verb or a noun, it is an adjective.

Predictor	Original model				Bootstrapped model				CI lower	CI upper	ΔR^2^
	β	Std. error	t	*p*	β	Std. error	Z	*p*			
**Intercept**	9.87	2.29	4.31	0	9.92	2.68	3.71	0	4.66	15.26	-
**LogFrequency**	-0.76	0.15	-4.93	0	-0.76	0.17	-4.37	0	-1.11	-0.42	16.3%
**Synonyms**	1.84	0.42	4.33	0	1.83	0.42	4.38	0	0.98	2.62	12.5%
**MutualInformation**	0.03	0.12	0.21	0.834	0.02	0.14	0.14	0.891	-0.26	0.27	0.0%
**Imageability**	-0.67	0.22	-3.02	0.003	-0.67	0.24	-2.78	0.005	-1.14	-0.19	6.1%
**Arousal**	-0.19	0.15	-1.25	0.215	-0.2	0.14	-1.41	0.158	-0.48	0.08	1.0%
**LogSenses**	-0.55	0.24	-2.25	0.027	-0.53	0.24	-2.18	0.029	-1	-0.04	3.4%
**LogAgeOfAcq**	-0.36	0.74	-0.48	0.629	-0.36	0.74	-0.48	0.629	-1.79	1.16	0.2%
**WordClass: Noun**	1.15	0.74	1.55	0.125	1.16	0.75	1.54	0.123	-0.24	2.72	1.6%
**WordClass: Verb**	0.38	0.54	0.7	0.486	0.4	0.46	0.87	0.386	-0.47	1.33	0.3%

The model shows a decent fit (N = 117, R^2^ = 0.34, *F*(9, 99) = 5.62, *p* < .0001) explaining about 34% of the variance of the lexical replacement rate. Crucially, the fit is significantly better than that of a model only including log Frequency and Word Class as predictors (χ^2^(6) = 247.42, *p* < .0001). The predictor statistics are shown in Table 3, which includes statistics of both the original and the bootstrapped model. The final column reports the ΔR^2^ of each predictor, which is a measure of the proportion of the variance of Lexical Replacement Rate explained by each individual predictor, over and above that of all other predictors in the model. ΔR^2^ was calculated with on the basis of the lmSupport package [48].

The results of the regression modeling replicate the results of Pagel et al. [2] in showing that word frequency is a strong predictor of the rate of lexical replacement. Frequency explains as much as about 16.3% of its variance after controlling for the influence of all other predictors: the more frequent a concept is, the less likely its primary lexical form is to be replaced, as shown by the negative sign of the beta coefficient.

**Fig 2:**
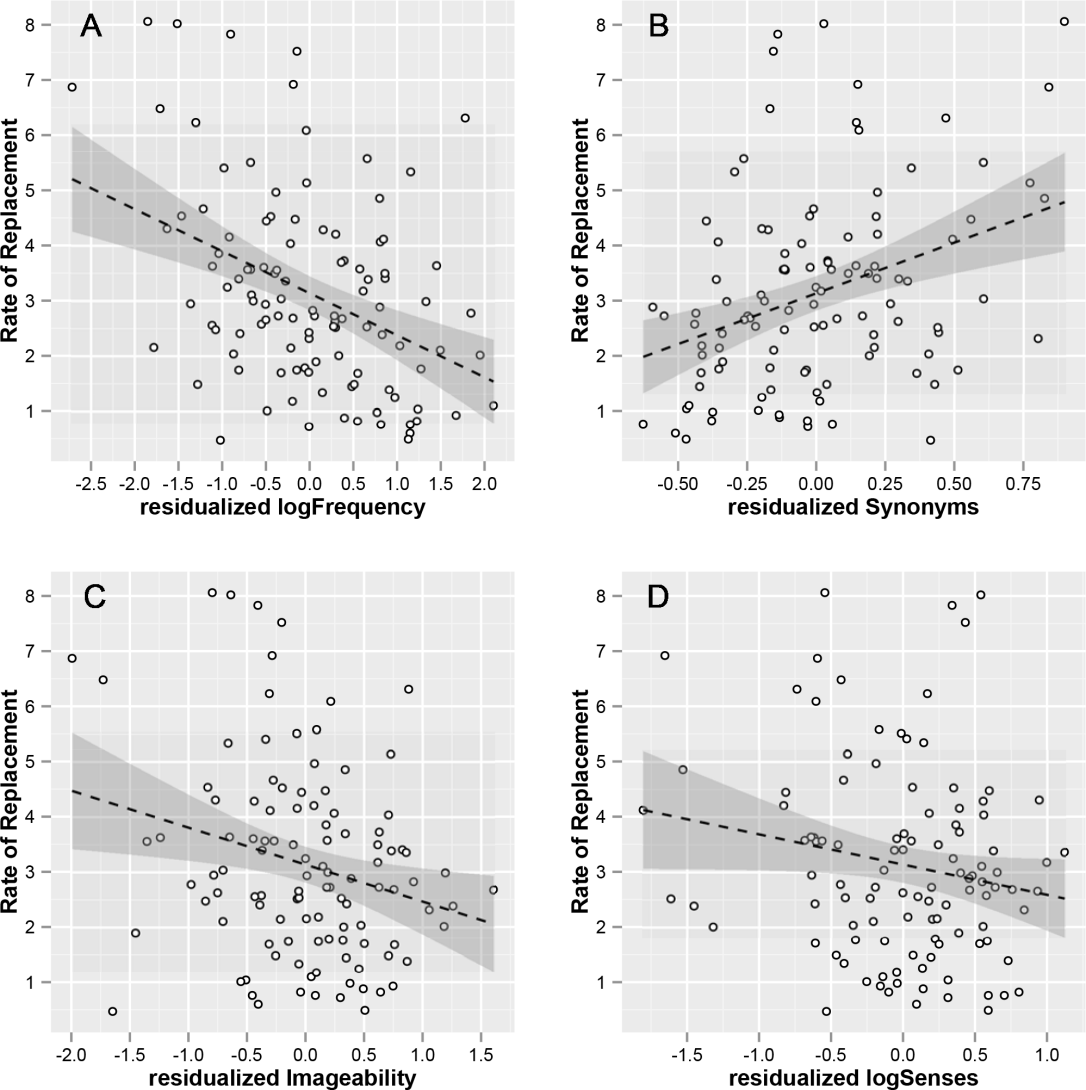
Scatterplots of the relationship between the rate of lexical replacement and logSenses. The left hand panel shows the relationship between Lexical Replacement Rate and LogSenses for three different levels of synonyms (Low: 0–0.65 mean synonyms; Medium: 0.65–1.1 mean synonyms; and High: 1.1–2.65 mean synonyms). The right hand panel shows the relationship between Lexical Replacement Rate and LogSenses when the average number of synonyms is not controlled for. Shaded areas represent 95% confidence intervals of the slopes of the regression lines.

Table 3 further shows that the imageability of a concept is also associated with a decrease in its lexical replacement rate. The predictor Imageability explains about 6.1% of the variance of the replacement rate. Although we did not find a significant correlation between Lexical Replacement Rate and LogSenses (see Table 2), there is a significant effect of LogSenses explaining close to 3.4% of the variance in Lexical Replacement Rate, when all other factors are controlled for: concepts whose primary forms have a greater number of senses show a weak, albeit significant, decrease in lexical replacement rate. This negative effect emerges when controlling for synonyms and is illustrated in Fig 2: when concepts are grouped with respect to their average amount of synonyms, a strong negative relationship between LexicalReplacementRate and LogSenses is found.

Importantly, the model also shows that the average amount of synonyms that are listed in synonym dictionaries for a concept is almost as strongly associated with the lexical replacement rate as frequency. The predictor Synonyms explains about 12.5% of the variance of the replacement rate. The greater the average amount of synonyms used for a concept is, the more likely its primary form is to be replaced. The regression model finally shows that the individual relationships between Lexical Replacement Rate and Mutual Information, (see Table 2) is in fact mediated by other variables in the model: once their influence is accounted for, the relationship disappears.

Likewise, and unlike Monaghan [5] we found no relationship between the Rate of Lexical Replacement and the Age of Acquisition when at least Frequency is controlled for. An additional regression analysis in which Rate of Lexical Replacement was regressed against Age of Acquisition and Frequency, also failed to find a significant influence of Age of Acquisition on Lexical Replacement Rate. We replicated Monaghan’s study successfully for that study’s entire 200 item data set, and then added a binary variable open class (all nouns, verbs, adjectives; 173 items) / closed class (the 27 remaining items) to that data set. The effect of Age of Acquisition on Lexical Replacement Rate is in fact driven by the difference between open and closed class words. We conducted separate analyses of Mohanagan's data across open and closed class words, respectively, and found no significant effect of Age of Acquisition in neither of them. More importantly, however, when the word class predictor is replaced by a predictor distinguishing open class and closed class words only, thereby controlling for their difference, analysis of the full data set finds no significant effect of Age of Acquisition is found.

The impact of arousal and word class was likewise not statistically significant.

**Fig 3:**
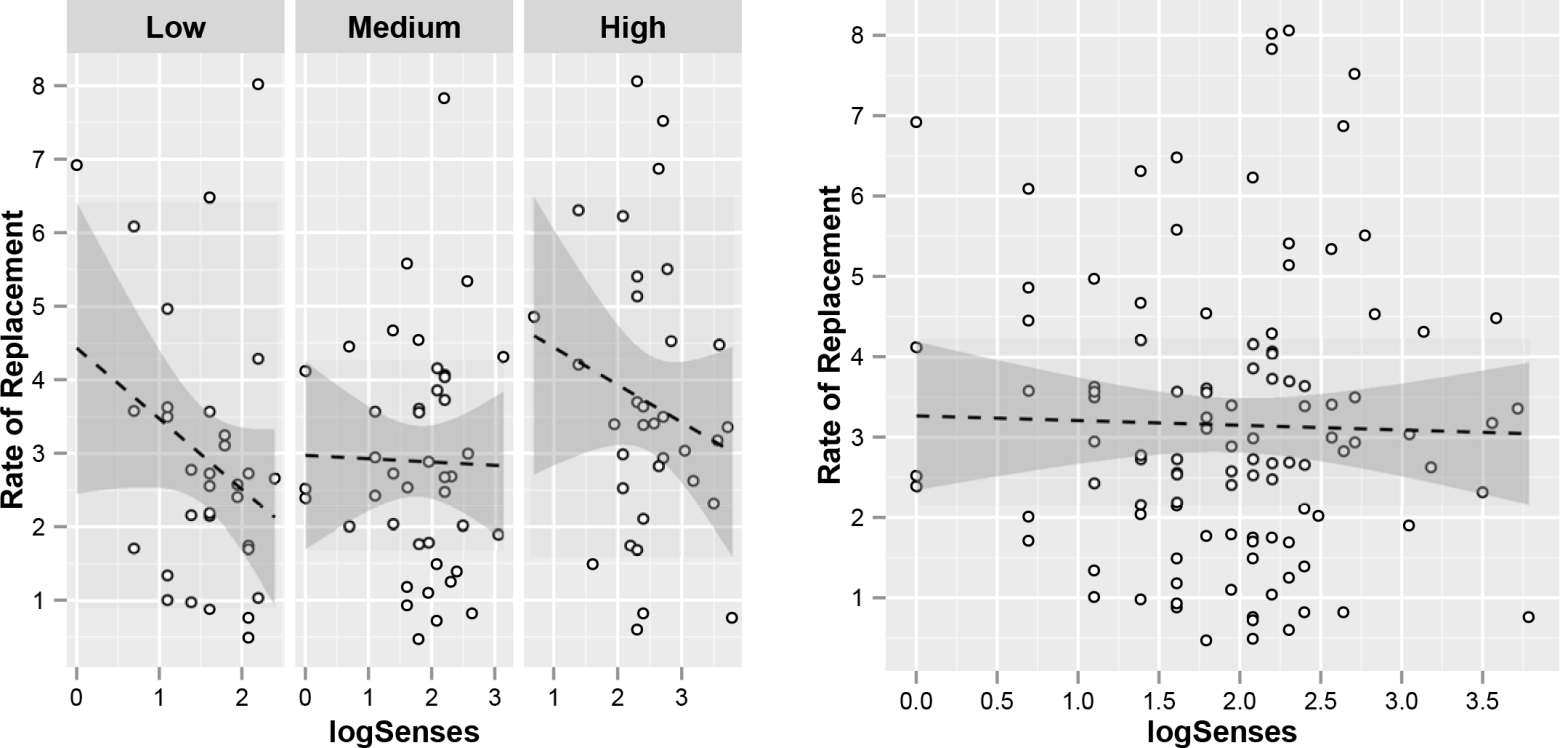
Scatterplots of the relationships between the rate of lexical replacement and (A) residualized log Frequency, (B), residualized Synonyms, (C) residualized Imageability and (D) residualized Senses. Shaded areas represent 95% confidence intervals of the slopes of the regression lines.

The significant relationships between LogFrequency, Synonyms, Imageability and LogSenses, on the one hand, and Lexical Replacement Rate, on the other, are illustrated in Fig 3. The figure illustrates the relationships between Lexical Replacement Rate and the aforementioned predictor variables while holding the influence of all other predictors constant. This is done by plotting the Lexical Replacement Rate against the residuals of each predictor variable regressed against all other predictors.

## Conclusions

In this paper, we have argued that function words and content words behave differently with respect to their rates of lexical replacement. Previous research into factors that influence the rate of lexical replacement has treated function words and content words together–by separating the two groups it becomes clear that they behave quite differently.

We have also shown that, in addition to frequency, the number of synonyms, the imageability and the number of senses associated with content word concepts predict the rate of lexical replacement of those concepts. The more synonyms that are used for a concept, the higher the lexical replacement rate of that concept is. We attribute this to the fact that availability of other semantically close words makes inferencing and replacement easier. We also found a negative relationship between the imageability (i.e. the ease of which the concept is depicted in the mind) of concepts and their lexical replacement rate. Concepts that are more easily imagined and pictured in the minds of the speakers therefore seem to undergo less word replacement. Hills and Adelman [31] note that concreteness (and, one might assume, also the similar measure of imageability) in a language may increase as it gains more second language speakers, which is something that English has done for the last few centuries, and this might contribute to why more concrete words are more resilient over time. We finally found a small negative relationship between the number of senses a concept has and its lexical replacement rate. We suggest that highly frequent and highly polysemous words (i.e., that are used in many different genres) are highly entrenched and therefore harder to replace.

Unlike Monaghan (2014) we found no relationship between the rate of lexical replacement and the age of acquisition when at least frequency is controlled for. We were also unable to show any significant contribution of the mutual information factor in the regression model, even though it is significantly correlated with the rate of lexical replacement on its own. Also, while some effect of arousal on the replacement rate of some taboo concepts seems indisputable, we could not show that this, when it is measured by arousal values derived from semantic differential experiments, was generally applicable.

A drawback of this study is that although the rate of lexical replacement is calculated on the basis of data from many different Indo-European languages, all of the independent variables are based upon data from either a few Germanic languages or English only. The reason for using mainly English data was practical since there are, at this time, no other languages where the substantial data collection efforts needed to assemble the data for the dependent variables have been completed. More data and further studies will make the results more reliably generalizable to other Indo-European languages. However, we believe that the shortcomings of the independent variables should work against the hypotheses rather than in favor of them: already in this limited study, strong correlations between the variables are seen even though idiosyncrasies with e.g. language particular homonyms should lead to more noise. Had the independent variables been based on data from a representative sample of Indo-European languages we expect stronger relationships between them and the rate of lexical replacement, not weaker.

To conclude, we have argued that there is reason to assess function words and content words separately with respect to their rate of lexical replacement and that, in addition to frequency, the semantic factors of synonyms, senses and imageability predict the rate of lexical replacement of content words.

## Supporting Information

S1 File Factors Affecting the Rate of Lexical Replacement Raw Data. (CSV)
